# Admixture, evolution, and variation in reproductive isolation in the Boechera puberula clade

**DOI:** 10.1186/s12862-018-1173-6

**Published:** 2018-04-25

**Authors:** Martin P. Schilling, Zachariah Gompert, Fay-Wei Li, Michael D. Windham, Paul G. Wolf

**Affiliations:** 1 Biology Department, Utah State University, 5305 Old Main Hill, Logan, UT, 84322 USA; 2 Ecology Center, Utah State University, 5205 Old Main Hill, Logan, UT, 84322 USA; 3 present address: Department of Ecology and Evolutionary Biology, University of Colorado, N211 Ramaley Hall, Boulder, CO, 80309 USA; 4 Department of Biology, Duke University, 266 Biological Sciences, Durham, NC, 27708 USA; 5 Boyce Thompson Institute, 533 Tower Rd, Ithaca, New York, 14853 USA; 6 Plant Biology Section, Cornell University, 237 Mann Dr, Ithaca, New York, 14853 USA

**Keywords:** Hybridization, Apomixis, Reproductive isolation, Speciation

## Abstract

**Background:**

Hybridization is very common in plants, and the incorporation of new alleles into existing lineages (i.e. admixture) can blur species boundaries. However, admixture also has the potential to increase standing genetic variation. With new sequencing methods, we can now study admixture and reproductive isolation at a much finer scale than in the past. The genus *Boechera* is an extraordinary example of admixture, with over 400 hybrid derivates of varying ploidy levels. Yet, few studies have assessed admixture in this genus on a genomic scale.

**Results:**

In this study, we used Genotyping-by-Sequencing (GBS) to clarify the evolution of the *Boechera puberula* clade, whose six members are scattered across the western United States. We further assessed patterns of admixture and reproductive isolation within the group, including two additional species (*B. stricta* and *B. retrofracta*) that are widespread across North America. Based on 14,815 common genetic variants, we found evidence for some cases of hybridization. We find evidence of both recent and more ancient admixture, and that levels of admixture vary across species.

**Conclusions:**

We present evidence for a monophyletic origin of the *B. puberula* group, and a split of *B. puberula* into two subspecies. Further, when inferring reproductive isolation on the basis of presence and absence of admixture, we found that the accumulation of reproductive isolation between species does not seem to occur linearly with time since divergence in this system. We discuss our results in the context of sexuality and asexuality in *Boechera*.

**Electronic supplementary material:**

The online version of this article (10.1186/s12862-018-1173-6) contains supplementary material, which is available to authorized users.

## Background

Hybridization is the interbreeding of individuals from genetically differentiated populations that are distinguished by multiple heritable characters [1, 2]. Hybridization can lead to offspring with reduced fitness, but it can also transfer alleles between species through introgression [3–6]. Such introgressed alleles represent a potential source of novel genotype combinations that might be adaptive in a new (or the old) environment. On the other hand, with high rates of gene flow and low levels of reproductive isolation (RI), introduced alleles could be transmitted in such a way that the genomes of locally adapted species with small population sizes (e.g. in alpine glacial refugia) could ultimately become extinct by being swamped by a different lineage [7, 8]. Thus, the incorporation of new alleles into existing lineages through hybridization (i.e. admixture) has varying effects on speciation and local adaptation, either slowing or accelerating the evolution of RI between populations through gene flow and recombination.

The study of admixture in natural populations has a long history [1, 2, 9–11], with a strong emphasis on discerning patterns in hybridization and its evolutionary role using a diverse array of techniques. Initially, morphological and behavioral characters were used to describe hybrid zones in animals [12–15] as well as plants e.g. [16, 17], based on the indicator of phenotypic intermediacy between parental species. As new techniques emerged, it became possible to combine morphological features with biochemical data, such as allozyme markers [18] as well as cytogenetic methods [19–21]. The pace of discovery in this field of inquiry increased significantly with the development of molecular markers [22–25] see [26, 27] for excellent reviews on the subject.

With the recent emergence of high-throughput sequencing methods, and more specifically the development of Genotyping-by-Sequencing (GBS) methods, the study of hybridization is once more experiencing a strong resurgence e.g [28–30]. We are now able to assess patterns of introgression on a genome-wide scale. The number of loci assayed across individual genomes has increased substantially, giving us the opportunity to study admixture at a scale that greatly exceeds the genomic resolution and statistical power obtained using previous marker methods [31–37]. We are now able to obtain more accurate estimates of admixture, detect more limited introgression, and measure variation in introgression among regions of the genome [30, 38, 39].

Certain groups of organisms are more prone to hybridization than others, and the flowering plant genus *Boechera* (Brassicaceae) is extraordinary in this regard. Early studies of this group as *Arabis* in [40–42] suggested that hybridization was common. More recent work has shown that *Boechera* comprises one of the most extensive and complex hybrid networks known, including 80+ sexual diploid taxa that have interacted to form over 400 distinct hybrid lineages containing two, three, or even four distinct genomes [43]. Hybridization spans the entire genus [44] and has occurred repeatedly and independently among many diploid species, resulting in hybrid taxa with high genetic diversity [45–47]. Sexual diploid *Boechera* species were found to be self-compatible, with low levels of heterozygosity and high levels of inbreeding [42, 47–49]. Hybrids were mostly found to be apomictic (reproducing via unfertilized seeds), highly heterozygous and it has been suggested that both obligate and facultative apomixis exist in this system [40, 50, 51].

Hybridization in *Boechera* appears to be strongly linked to the occurrence of gametophytic apomixis (Taraxacum-type diplosporous apomixis [52]), where meiosis I fails, and meiosis II results in the formation of two (rather than four) megaspores that are genetically very similar (often identical) to the sporophyte that produced them. One of these two cells degenerates, leaving the other to undergo three mitotic divisions to form a megagametophyte [42, 47–49, 52–56].

Over the last two decades, the genus *Boechera* has been intensively studied in regards to patterns of genomic architecture [57–60], local adaptation and speciation [61–66], hybridization and polyploidy as well as the origin and control of apomixis [45, 51, 53–55, 67–69], and population genetic differentiation of natural populations [49, 70]. The breadth of ongoing work, coupled with known high levels of inbreeding in *Boechera* species and its relatively close relationship to *Arabidopsis*, have made the genus a valuable model system for studies of evolution and ecology see also [71]. But to fully realize the potential of this model system, we need to better understand the patterns of admixture and reproductive isolation that have contributed to its evolution.

Our goal is to build on this foundation by assessing genome-wide patterns of hybridization and resulting admixture while clarifying the evolutionary relationships of one well-supported clade, the *Boechera puberula* group. We focus on tests for historical and contemporary hybridization (via the identification of hybrid or admixed individuals) rather than ancient introgression. As originally defined by [44], the *puberula* clade included five sexual diploid species: *B. lasiocarpa*, *B. puberula*, *B. retrofracta*, *B. subpinnatifida*, and *B. serpenticola*. The single specimen referred to as *B. retrofracta* in this earlier phylogenetic analysis [44] has subsequently been reassigned to *B. exilis*, with the epithet *retrofracta* applied to a different clade (Windham et al. unpubl.).

In this study, we attempt to 1) assess the evolutionary placement of the *Boechera puberula* group, a monophyletic clade within the large genus *Boechera* (Brassicaceae), and 2) estimate admixture proportions within these species to assess patterns of gene flow and levels of RI on a genome-wide scale. When taxa overlap and have opportunities for gene flow, the presence versus absence of hybrids can be seen as evidence for the strength of RI. Jointly, these analyses will form the basis for future work on speciation in the group.

## Methods

### Data collection and DNA extraction

**Fig. 1:**
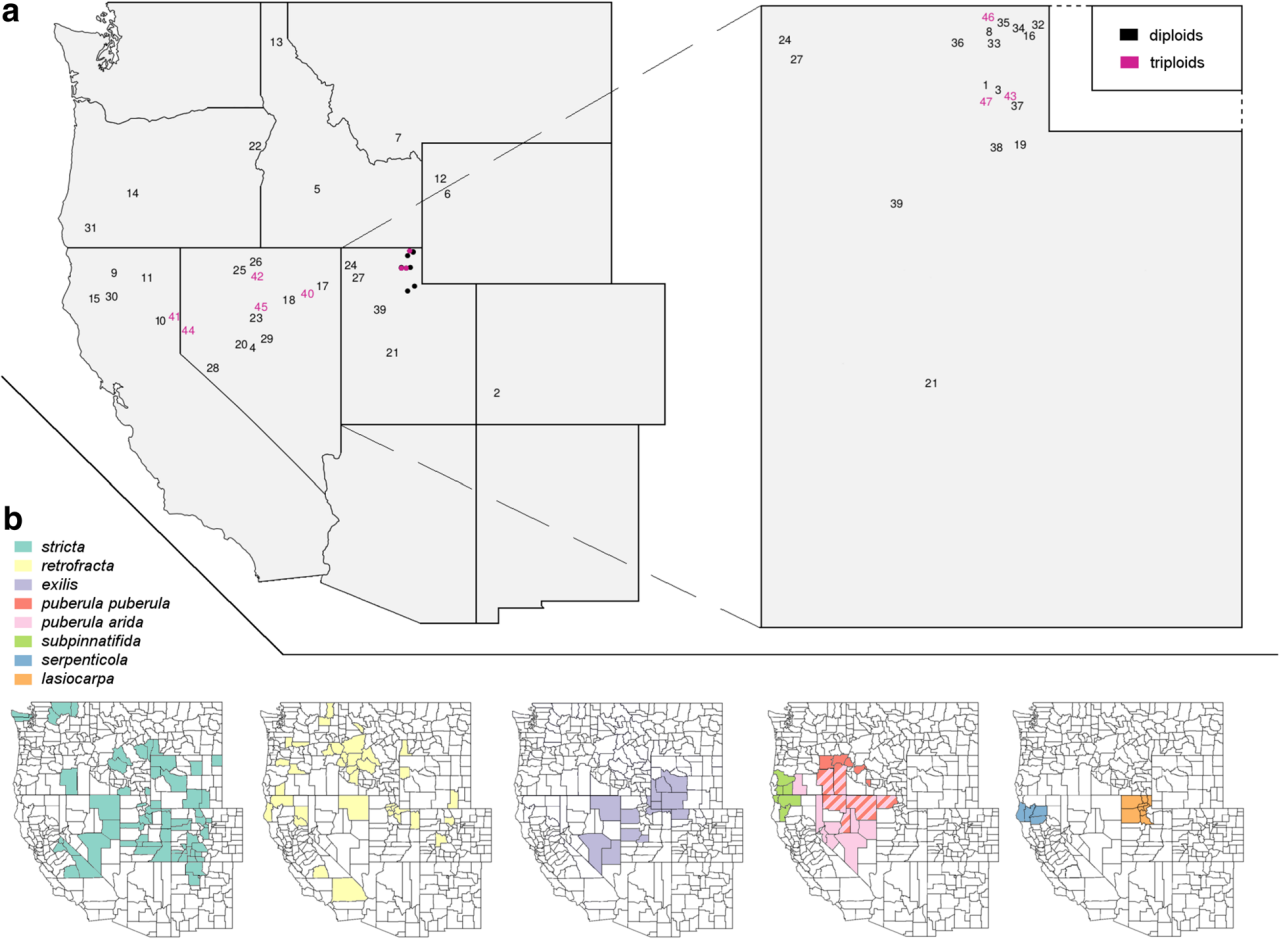
Sampling locations and distribution ranges of select *Boechera* taxa in western United States. **a** Sampling locations of diploid and triploid *Boechera* populations including blowup of UT (see locality numbers and nominal taxa in Table 1). Note that we added a small amount of noise to GPS coordinates, in order to make all of the the locality numbers visible. Exact geographic coordinates are provided in Table 1. **b** Documented distributions of *Boechera* species across the western United States. Maps include only sexual diploids identified by their epithets. Note that these county-level distributions are based solely on specimens whose identification has been confirmed by microsatellite studies [56]. Both *B. retrofracta* and *B. stricta* have wider distributions across North America

We extracted DNA from leaf tissue of 107 individuals from 47 localities in spring and early summer of 2013 (see Fig. 1 and Table 1 for a list of taxa and sampling localities). Leaf tissue collections made by the senior author (identified by the prefix “MS”) were immediately stored in silica gel and voucher specimens are accessioned in the Intermountain Herbarium (UTC). The 23 samples without the prefix “MS” came from air-dried herbarium specimens deposited at the herbaria indicated in Additional file 1: Table S1. DNA was extracted from leaf tissue following the CTAB protocol described in [55].

**Table 1:** Locality information for Fig. 1, sample numbers for Figs. 2, 3, and 4 and Additional file 1: Figure S3 as well as ploidy and nominal taxa which were both determined from microsatellite data (*Continued*)

Locality	Samples	Locality	Longitude	Latitude	Ploidy	Nominal taxon
1	1-3,8	Grizzly Peak, UT	-111.97	41.41	2	*B. stricta*
2	4	La Plata, CO	-108.02	37.44	2	*B. stricta*
3	5	Weber, UT	-111.59	41.41	2	*B. stricta*
4	6	Nye, NV	-117.35	38.95	2	*B. stricta*
5	7	Custer, ID	-114.65	43.86	2	*B. stricta*
6	9	Teton, WY	-110.52	43.85	2	*B. stricta*
7	10	Madison, MO	-111.96	45.56	2	*B. stricta*
8	11,13,14,25	Steep Canyon, UT	-111.6	41.97	2	*B. retrofracta x stricta*
9	12,24,26	Deadfall Lake, CA	-122.52	41.33	2	*B. retrofracta x subpinnatifida*
10	15,18,19	Little Volcano, CA	-120.89	39.86	2	*B. retrofracta*
11	16,21,47,75	Hat Creek, CA	-121.41	40.7	2	*B. retrofracta*
12	17	Park, WY	-110.57	44.41	2	*B. retrofracta retrofracta* (sexual)
13	20	Mineral, MO	-115.7	47.45	2	*B. retrofracta retrofracta* (sexual)
14	22	Deschutes, OR	-121.56	43.67	2	*B. retrofracta retrofracta* (sexual)
15	23	Humboldt, CA	-123.65	40.48	2	*B. retrofracta retrofracta* (sexual)
16	27,31,33,34	Bear Lake Summit, UT	-111.47	41.93	2	*B. exilis x retrofracta*
17	28,29,30,32	Wells, NV	-114.57	41.08	2	*B. exilis x retrofracta*
18	35	Elko, NV	-115.08	40.68	2	*B. exilis*
19	36	Summit, UT	-111.4078	40.7753	2	*B. exilis*
20	37	Nye, NV	-117.54	38.97	2	*B. exilis*
21	38	Millard, UT	-112.27	38.95	2	*B. exilis*
22	39	Baker, OR	-117.11	44.7	2	*B. puberula puberula*
23	40,41,48,49	Water Canyon, NV	-116.71	40.64	2	*B. puberula*
24	42	Box Elder, UT	-113.94	41.77	2	*B. puberula puberula*
25	43,45,46	Lye Creek, NV	-117.54	41.69	2	*B. puberula*
26	44	Humboldt, NV	-117.55	41.67	2	*B. puberula puberula*
27	50	Box Elder, UT	-113.69	41.53	2	*B. puberula arida*
28	51	Mono, CA	-119.13	38.36	2	*B. puberula arida*
29	52	Lander, NV	-117.37	39.24	2	*B. puberula arida*
30	53-58	Bully Choop Mtn, CA	-122.94	40.65	2	*B. serpenticola*
31	59-62	Rogue River, OR	-123.53	42.55	2	*B. subpinnatifida*
32	63	Rich, UT	-111.46	41.92	2	*B. lasiocarpa* (holotype)
33	64,65	Cache, UT	-111.66	41.91	2	*B. lasiocarpa*
34	66-71	Logan Canyon Sinks, UT	-111.48	41.93	2	*B. lasiocarpa*
35	72-74	Steam Mill Peak, UT	-111.61	41.95	2	*B. lasiocarpa*
36	76	Box Elder, UT	-111.98	41.39	2	*B. lasiocarpa*
37	77	James Peak, UT	-111.78	41.38	2	*B. lasiocarpa*
38	78	Salt Lake, UT	-111.72	40.63	2	*B. lasiocarpa*
39	79	Tooele, UT	-112.62	40.48	2	*B. lasiocarpa*
40	-	Angel Lake, NV	-115.07	41.02	3	*B. exilis x puberula x retrofracta*
41	-	Frenchman Lake, CA	-120.18	39.87	3	*B. retrofracta x sparsiflora* (2:1)
42	-	Indian Creek, NV	-117.55	41.65	3	*B. exilis x retrofracta x sparsiflora*
43	-	James Peak, UT	-111.78	41.38	3	*B. retrofracta x lasiocarpa* (2:1)
44	-	Peavine Peak, NV	-119.93	39.59	3	*B. puberula arida x subpinnatifida* (2:1)
45	-	Shoshone Mtns, NV	-116.86	40.42	3	*B. exilis x puberula x retrofracta*
46	-	Steam Mill Peak, UT	-111.61	41.95	3	*B. lasiocarpa x retrofracta x stricta*
47	-	Willard Peak, UT	-111.97	41.39	3	*B. lasiocarpa x lemmonii x stricta*

Complete locality and sample information can be found in Additional file 1: Table S1

### Microsatellite markers for determination of ploidy and nominal taxa

We generated microsatellite data to determine ploidy and assign plants to nominal species. Microsatellite markers were genotyped at 15 loci using the multiplex polymerase chain reaction (PCR) protocol described in [55]. We then determined the size of amplicons on an Applied Biosystems 3730xl DNA Analyzer, and alleles were scored using GeneMarker version 2.6.2 (Softgenetics, State College, PA, USA). We further inferred the ploidy level of each sample by determining the maximum number of microsatellite alleles at each locus, which has been shown to be an accurate proxy for chromosome counts in *Boechera* [43, 55]. We then identified nominal taxa based on a dataset containing roughly 4400 individuals, representing all currently known sexual diploid *Boechera* species [43, 55, 72].

### Genotyping-by-Sequencing library

We generated Genotyping-by-Sequencing (GBS) data to resolve the evolutionary placement within *Boechera* and infer admixture proportions. Reduced-complexity, double-digest restriction fragment-based DNA libraries were prepared for the same DNA samples, following [30]. The restriction-fragment library preparation method generally yields high numbers of loci through the use of high-throughput sequencing platforms, as compared to traditional molecular markers. For studies of admixture, we can thus expect to achieve a higher resolution across individuals’ genomes. The GBS libraries were sequenced in one lane at the University of Texas Genomic Sequencing and Analysis Facility (Austin, TX, USA) on the Illumina HiSeq 2500 platform. We used custom python and perl scripts [73] to parse the sequences for individual barcodes and split them by individual. Each individual was aligned to the *B. stricta* genome assembly [74] using bwa aln & samse version 0.7.5 [75]. We allowed for a maximum edit distance of 5, with a read trimming parameter of 10, the seed set to 20 and a maximum edit distance in the seed of 2. We further used samtools version 0.1.19 [76] to create bam files from the resulting alignments. Polyploids were excluded, because subsequent analyses only permit the use of diploid individuals. In total, we considered 79 diploid individuals for further analyses (see Table 1).

We identified single nucleotide variants (SNVs) using GATK version 3.5 [77] with ploidy set to diploid. Using the Unified Genotyper in GATK, we set heterozygosity for prior likelihood calculation per locus to 0.001, and ignored sequences with mapping quality < 20. We further set the minimum phred-scaled confidence threshold for variants to be called to 50. The resulting variants were further filtered to contain only variants with at least 128 sequences, at least 4 sequences with the alternative allele, and we only kept the genetic variants at nucleotide sites where we had data for at least 80% of the sampled individuals and only one alternative allele. Additionally, only variants with minimum phred-scaled mapping quality of 30 and a minor allele frequency < 0.05 were retained, in order to keep only common variants.

### Evolutionary history and genetic structure of the *B. puberula* group

To assess the evolutionary history of the *B. puberula* group, we performed clustering of individuals based on genotype estimates for the common SNVs. We used the posterior mean genotype as a point estimate for genotypes based on the posterior genotype probabilities for eight putative source taxa, as obtained by entropy [30] (see below). A mean genotype is the mean of the posterior distribution and as such is a non-integer point estimate of the number of alleles at a given locus, ranging from zero to two (with 0: homozygous for reference allele; 1: heterozygous, and 2: homozygous for the alternative allele). Because we are using SNVs, we are not dealing with continuous or contiguous stretches of DNA sequences. On the contrary, we use only variable sites that were concatenated into a string of variants for each individual. Since assuming a standard model of sequence evolution might not be accurate under these circumstances, we used distance methods instead. Consequently, branch lengths of the resulting trees can not be directly related to substitution rates, as they represent the distance matrix across individuals and SNVs, and we could not infer the timing of diversification between these taxa see also [78]. We created a neighbour-joining (NJ) tree based on a matrix of pairwise distances of the number of sites that differ between each pair of concatenated SNV sequences. The NJ tree was constructed by using the ape package (version 3.4) [79] in R and the distance matrix was constructed with the dist.dna function.

The common SNVs were analysed for population genetic structure and admixture using entropy, which is described in [30]. This model is very similar to the correlated allele frequency admixture model in structure [80], but here, sequence coverage, sequencing error, and alignment error are explicitly included in the model. Such a procedure has been demonstrated to decrease bias when compared to called genotypes [39]. The output of entropy includes admixture proportions, genotype probabilities for all individuals at all loci and credible intervals for all estimated parameters. We performed the analysis with entropy for numbers of k of 2 to 16 putative clusters, with 6 chains for each k. In order to minimize the computional time required for entropy runs, we estimated initial mean genotypes for each individual and locus from the genotype likelihoods by using the expectation-maximization algorithm described in [38]. These mean genotype estimates were used to calculate starting values of admixture proportions with the discriminant analysis of principal components (dapc) [81] function in the R package adegenet [82, 83] for each respective number of clusters.

The software entropy (like structure) uses multilocus genotype data to estimate admixture proportions given a number k of source populations. Due to the inherent stochastic nature of the MCMC sampling algorithm, results will likely not be exactly the same between repeated runs. Furthermore, if the number of sampling iterations is not sufficiently large to reach convergence, repeated runs are likely to differ significantly. Thus, it is advisable to choose appropriate numbers of iterations and to perform cross-validation between multiple runs of the same k [84, 85]. We assessed convergence and mixing of chains using the Gelman-Rubin diagnostic [86] in coda [87] and created the corresponding barplots of admixture proportions for all k clusters (source populations) in R [88]. The optimal number of clusters was determined by comparing the deviance information criteria (DIC) for respective chains across all k clusters.

A second analysis was run in entropy to quantify the extent to which hybrids were heterozygous for ancestry from different lineages (i.e., from different inferred source populations). This was done using the Q model, which explicitly models the proportion of each individual’s genome where both gene copies come from the same versus different source populations (this is an extension of the standard model used to infer admixture proportions) [30]. For this analysis, we only considered k = 2. Thus, our focus here was on hybrids between the *B. puberula* clade and either *B. stricta* or *B. retrofracta* (these were the groups distinguished by k = 2). We ran five chains each with a 5000 iteration burnin, 20,000 post-burnin steps and a thinning interval of 15. These runs were also seeded with starting values estimated from dapc.

We extracted the estimated posterior genotype probabilities for eight clusters along with 95% credible intervals. These genotype probabilities were used to construct a covariance matrix of mean genotypes across all common SNVs (14,815) for all 79 diploid individuals. We used principal component analysis (PCA) to summarize the genotype data based on the centered but not scaled genotype estimates using the prcomp function in R. Additionally, the estimated mean genotypes, obtained from entropy, were used to perform the assessment of evolutionary placement as mentioned above.

As a further way to assess the evolutionary history and verify admixture events within the *B. puberula* group, we used treemix version 1.13 [89]. With this method, we can formally test for the presence of splits and mixtures in the history of our sampled populations. A bifurcating tree is first fit based on the population allele frequency correlation matrix. Migration edges are then added to improve the fit of the model; this creates a population graph or network. Across the 79 individuals with 14,815 variants, we inferred a population graph of said samples with 0 - 11 migration events, rooted with *B. stricta*, and we calculated the variance in population relatedness explained by the treemix model to quantify model fit (Additional file 1: Figure S1). For this analysis, individuals were grouped into populations/species based on the taxon assignments from the microsatellite data set (this is the current standard for taxonomy in this group of organisms). We did however drop a single individual from this analysis, sample 47, which had strongly conflicting assignments based on the microsatellite data (*B. retrofracta*) and its admixture proportions inferred from the GBS data (nearly pure *B. puberula*, see Results). Other individuals with more minor conflicts (e.g., evidence of admixture only from the GBS data) were retained in the nominal group defined by the microsatellites, and thus by current taxonomy for this group.

## Results

### Microsatellite markers for determination of ploidy and nominal taxa

Based on the maximum number of microsatellite alleles at each locus, we inferred that 79 of the sampled individuals from 39 localities were diploid, and 28 individuals from eight localities were triploid (see Fig. 1 and Table 1). Using the analytical tools and comparative data provided by [43], we determined that 64 of the diploid samples represented known sexual taxa. Our sampling included all species assigned to the *B. puberula* group by [44] as well as the two most widely distributed *Boechera* species, *B. retrofracta* and *B. stricta* (Fig. 1). The other 15 diploid individuals were inferred to be hybrids, produced by crosses between *B. retrofracta* and three other taxa (*B. exilis*, *B. stricta*, and *B. subpinnatifida*). All 28 triploid individuals showed evidence of hybrid origins involving two or, more often, three genomes (9 and 19 samples respectively; see Table 1 and Additional file 1: Table S1).

### Genotyping-by-Sequencing library

Our data comprised 57.8 ×10^6^ reads from 79 individuals, with a median of 677,138 reads per individual. We detected a total of 141,846 variants, and after quality filtering, we obtained 14,815 common high-quality variants (mean coverage per SNV per individual = 16.41, sd = 11.97). A randomly chosen set of 10% of the samples were replicated in the GBS library, which were further checked for consistency, and no deviations could be detected. Although we found some variation in the mean percentage of reads aligned among the sampled taxa, the distribution of mapping rates (across individuals) was largely overlapping among species (see Additional file 1: Figure S2). Moreover, when excluding known hybrids (based on the microsatellite data), we found no evidence that mapping rates were associated with variation in admixture proportions (see Additional file 1: Figure S3). Similarly, we found no evidence that variation in levels of admixture within taxa was associated with variation in mapping rates (e.g., within *B. retrofracta*, mapping rates for samples 15, 16 and 18 did not differ significantly from those for 17 and 19 −23; P = 0.373 from a permutation test). These results suggest that reference sequence bias is unlikely to have been a substantial issue for inferences from this data set.

### Evolutionary history and genetic structure of the *B. puberula* group

**Fig. 2:**
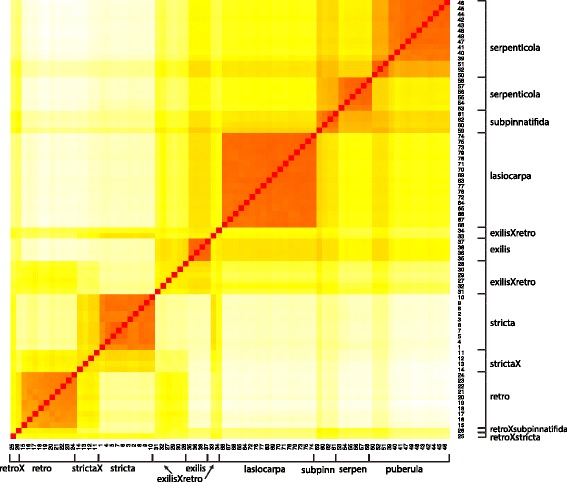
Heatmap of distance matrix between all 79 diploid individuals sampled. Numbers correspond to ids in Table 1 and Additional file 1: Table S1. Red cells indicates most similar comparisons, white is most different, and yellow and orange are intermediate. Broad taxon assignments are indicated. Note that “strictaX” refers to *B. stricta* x *B. retrofracta* (11,13,14) and *B. stricta* x *B. subpinnatifida* (12). Samples 47 and 75 (labeled *puberula* and *lasiocarpa*, (respectively, in figure) were initially assigned as *B. retrofracta*

**Fig. 3:**
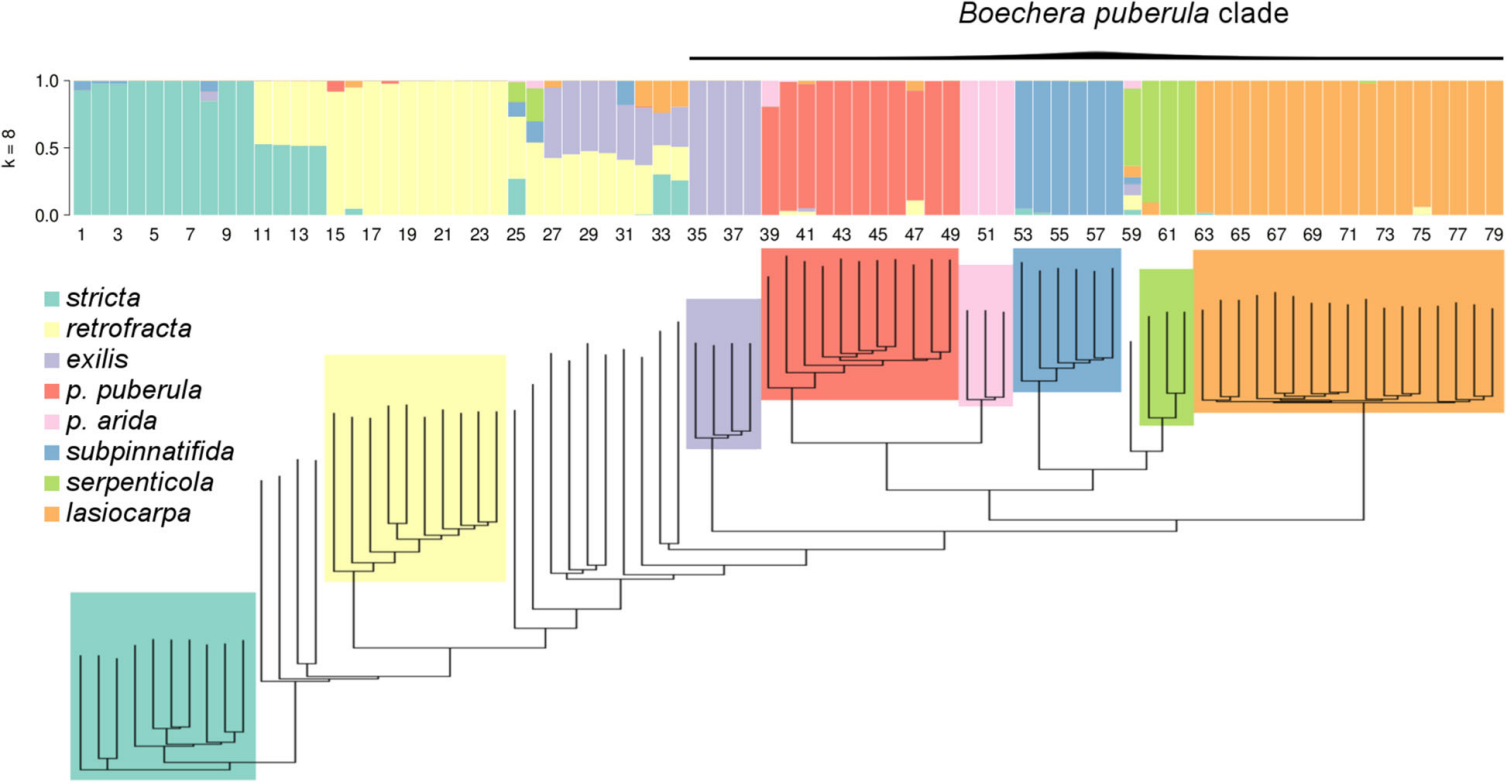
Admixture proportions for eight taxa and neighbor-joining tree of *B. puberula* group members as well as *B. stricta* and *B. retrofracta* based on common variants (n= 14,815)

Trees inferred from the GBS data were generally well-resolved by different methods of visualization. A heat map of genetic distances shows the general high similarity of samples within taxa, and between admixed samples and their parental taxa (Fig. 2). The neighbor-joining tree obtained from the estimated posterior genotype probabilities for eight source taxa shows clear differentiation of all putative sexual taxa, and was generally consistent with the heat map (Fig. 3). As suggested by the microsatellite data [43], *Boechera puberula*, as currently circumscribed, comprises two distinct monophyletic lineages, with the typical taxon (“*B. puberula puberula*”) occupying the northern part of the range and “*B. puberula arida*” replacing it to the south (Fig. 1). The clade formed by these two taxa is, in turn, sister to a lineage comprising *B. serpenticola* and *B. subpinnatifida* (Fig. 3). The latter form distinct, monophyletic groups. A monophyletic assemblage consisting of all 17 samples of *B. lasiocarpa* is sister to the *puberula*/*serpenticola*/*subpinnatifida* lineage, and this larger clade is, in turn, sister to *B. exilis*. Although hybrids are not well accommodated by the bifurcating tree model, their inclusion in the phylogenetic analyses reveals an interesting pattern, where the individuals identified as hybrids are placed between the respective parental species. The next sexual diploid lineage proximal to the lineage outlined above is a monophyletic grouping of all ten samples of *B. retrofracta* (Fig. 3). Said samples are separated on the tree by a grade consisting of ten accessions, all of which represent hybrids between *B. retrofracta* and members of the larger clade. Similarly, the branch between *B. retrofracta* and the proximal sexual diploid *B. stricta* is occupied by a grade of four samples, all of which are identified as *retrofracta x stricta* hybrids.

**Fig. 4:**
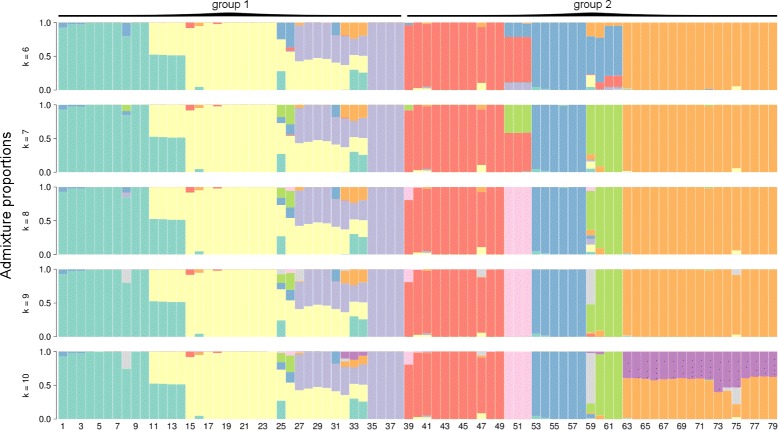
Admixture proportions based on 14,815 common variants. Each bar represents Bayesian point estimates of admixture proportions for each respective individual, and thus the proportion of inheritance of each genome to the respective species. Results of 6-10 presumed source species are shown here, with k = 8 being the best model based on DIC values. Groups 1 & 2 denote groups with differential admixture patterns. Results for k of 2 through 16 are shown in Additional file 1: Figure S4

Genetic variation of the common SNVs was best explained by an admixture model with eight source populations (DIC = 1.348 ×10^5^ compared to 1.357 ×10^5^ with k = 7), in accordance with the findings of [44] (Fig. 4). Gelman-Rubin diagnostics across all estimated admixture proportions and eight source populations indicated convergence of chains (median scale reduction factor = 1.057, mean = 1.082). The absolute difference between the lower and upper credible intervals of estimated admixture proportions across all individuals and source populations, as obtained from entropy, had a median of 5.65 ×10^−6^ (mean = 0.0102). Given the narrow width of these credible intervals (and thus low-level of uncertainty in the admixture proportions) we focus on the point estimates of the admixture proportions, which are given by the mean of the posterior distribution. Additional file 1: Figure S4 shows the admixture proportions for all putative source populations considered in this study, with k ranging from 2 to 16.

As expected from prior evidence of extensive hybridization in the genus *Boechera*, we found multiple individuals with mixed ancestry in the represented samples (see Fig. 3). In particular, most of the admixed individuals showed similar levels of admixture regardless of the number of source taxa assumed (Fig. 4). In the lower ranges of k in Additional file 1: Figure S4, we found that clusters differentiated individuals into the nominal taxa of *B. stricta*, *B. retrofracta* and *B. exilis*, with considerable admixture between those taxa. The *puberula* group was differentiated into three groups at k = 6, comprising *B. lasiocarpa*, *B. puberula* in the broad sense, and *B. subpinnatifida*/*B. serpenticola*. Comparing the admixture proportions at k = 8 (Fig. 3) with the nominal taxa based on microsatellite data (Table 1), we find congruence between the two datasets. Of the 79 samples included in both analyses, 62 (79%) showed admixture proportions deviating from expectations based on microsatellites by no more than 5%. In another 12 samples (15%), admixture proportions deviated from microsatellite-based expectations by 6 −30%. Five samples (6%) yielded admixture proportions that were strongly at-odds with microsatellite-based identifications.

Estimates of inter-lineage ancestry from the ‘Q’ model in entropy showed that individuals with mixed ancestry were heterozygous for ancestry at many loci (*Q*_12_> 0.2, that is heterozygosity for ancestry at greater than 20% of loci, for most samples; Additional file 1: Figure S5). Most (three of four) hybrids between *B. stricta* and *B. retrfracta* were advanced back-crosses to *B. retrofracta* (they had maximal ancestry heterozygosity given their admixture proportion, which means at least one of their parents was not admixed). Several individuals classified as *B. retrofracta* also appeared to be back-crosses, but with less overall ancestry from *B. stricta* (see the yellow dots on the line in Additional file 1: Figure S5). Ancestry estimates for the *B. retrofracta* ×*subpinnatifida* hybrids were consistent with one being a back-cross to *B. retrofracta* and one being a possible F1 or other early generation hybrid (e.g., F2, F3, etc.). Finally, *B. retrofracta* ×*B. exilis* hybrids spanned a range of inter-lineage ancestry suggesting a variety of late generation hybrids along with a likely back-cross to *B. exilis* and perhaps a F1 or other early generation hybrid (open square near the top of the triangle in Additional file 1: Figure S5).

When considering the model-free approach to describing genetic variation across samples, the majority (95.2%) of genetic variation was explained by the first three principal components (PCs), with the first two PCs accounting for 83.2% of the variation (see Fig. 6). Interestingly, the first principal component (with 69.7% explained variation) separated the two lineages *B. retrofracta* and *B. stricta* from the remaining lineages considered here (*B. puberula puberula*, *B. puberula arida*, *B. exilis*, *B. serpenticola*, *B. subpinnatifida*, and *B. lasiocarpa*), with admixed individuals positioned between the two groups. Principal component 2 (13.5% explained variation) separated *B. stricta* from *B. retrofracta*, with admixed individuals having intermediate scores on this PC. Additionally, PC 2 separated the *puberula* group lineages from each other, with *B. exilis* located between the other members of the *puberula* group. On principal component 3, we can see a similar pattern, where the spread between the members of the *puberula* group was wider, yet *B. exilis* was positioned between those. Furthermore, on PC 3, *B. stricta* and *B. retrofracta* were placed on opposite ends of the spectrum. None of the admixed individuals showed extreme PC scores on any of the 3 PCs, but they all showed intermediate values. On PC 2, and more strongly on PC 3, we found *B. lasiocarpa* individuals to be somewhat distinct from the remainder of the *puberula group* taxa.

**Fig. 5:**
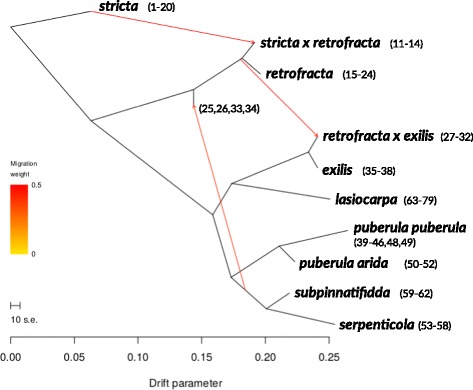
Graph structure inferred by treemix with four migration or admixture events for 79 diploid *Boechera* specimens, rooted with *B. stricta*. Arrows are colored by migration weight and branch lengths are proportional to genetic drift

In the population graph treemix analyses, the rooted population graph without admixture (i.e. migration edges) explained 90% of the variance in population relatedness (Additional file 1: Figure S6). When increasing the number of migration (or admixture) events, the variance explained increased rapidly. Here, we focus on the population graph with three admixture events (Fig. 5), which explained 99.8% of the variance in population allele frequency correlations (see Additional file 1: Figure S1 for graph structures for additional migration events). This population graph shows evidence of admixture between members of the *B. puberula* group and both *B. stricta* and *B. retrofracta*, consistent with the admixture proportion estimates from entropy.

**Fig. 6:**
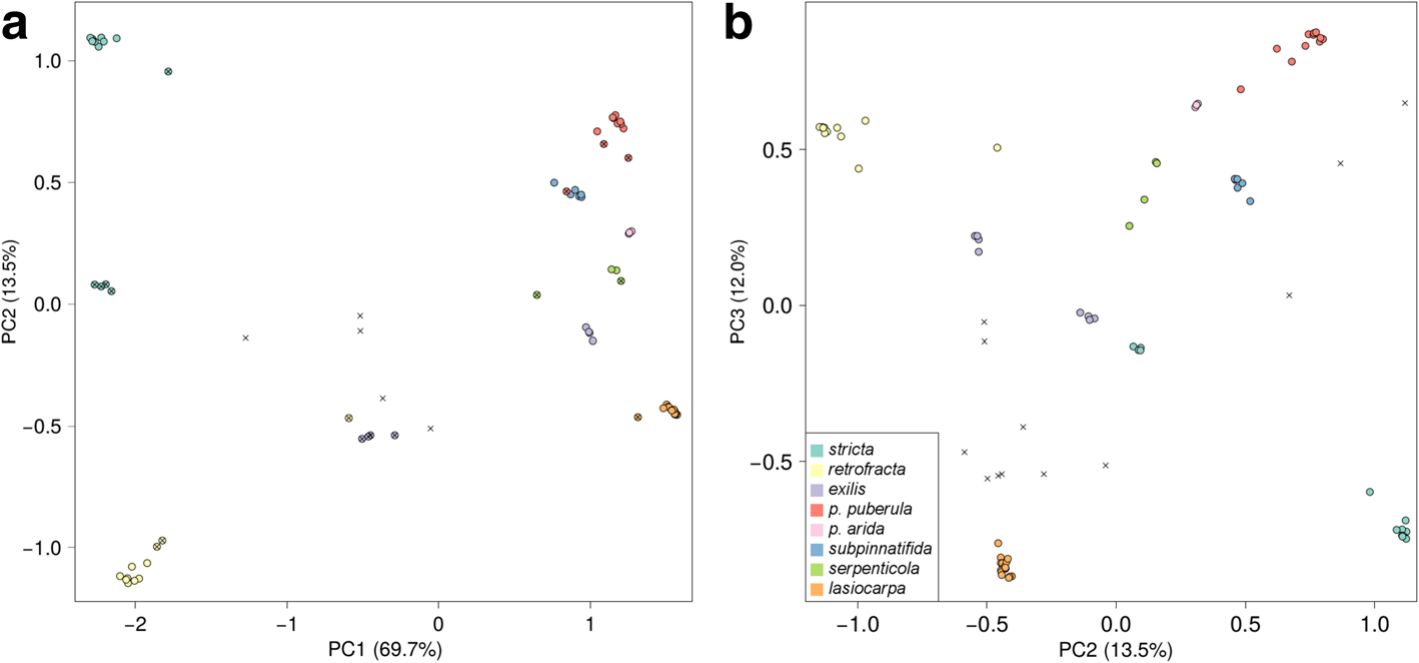
Statistical summary of genetic variation based on principal component analysis of 14,815 common variants, with **a** PC1 against PC2, and **b** PC2 against PC3, with colors assigned to individuals with posterior estimated admixture proportions above 95%, where the legend in **b** is used for both figures. Hybrids with less than 95% admixture proportions are drawn as crosses, and hybrids with more than 50% admixture proportions from a particular species are drawn as colored circles in the respective color with a cross

## Discussion

In this study, we described the evolutionary relationships and patterns of admixture among eight of the 80+ sexual diploid members of the genus *Boechera*. In order to achieve high resolution on a genome-wide scale, we used a GBS approach that allowed us to examine genetic variation across 14,815 common high-quality SNVs in the taxa studied. Our results support the monophyly of a cluster of six taxa, closely approximating the *B. puberula* species group first identified by [44]. In the neighbour-joining tree (Fig. 3), *B. puberula* (which forms two discrete clusters referred to as *puberula puberula* and *puberula arida*) is sister to a clade with two somewhat more divergent taxa, *B. subpinnatifida* and *B. serpenticola*. Sister to these core *puberula* taxa are the more distantly related members of the group, *B. lasiocarpa* and *B. exilis* (Fig. 3). Our results are congruent with the parsimony analysis of DNA sequences from seven nuclear loci [44], but show much improved resolution of species relationships within the group.

Two nomenclatural adjustments are necessary to allow direct comparison between our NJ tree and the previously published cladogram of the *B. puberula* group. With reference to Fig. 4 of [44], a single accession, identified as “*B. subpinnatifida*”, has been shown, based on recent microsatellite analyses, to represent *B. puberula puberula*, and the two specimens called “*B. puberula*” are now classified as *B. puberula arida* [43]. With these annotations, the two evolutionary trees of the *B. puberula* species group are seen to be consistent at the species level. The more extensive sampling achieved in this study (incorporating more loci, more individuals, and all six taxa) significantly improves our understanding of relationships within the group. In the tree from Alexander et al. [44], the only resolution within the *B. puberula* group involved the strong association between the two *B. serpenticola* accessions, the equally strong association of the two *B. puberula* (now *puberula arida*) samples, and a sister relationship between the latter and what is now referred to as *puberula puberula*. These two clades formed a polytomy with *B. lasiocarpa* and *B. exilis*. Our tree, on the other hand, is fully resolved (Fig. 3), with an *arida*/*puberula* clade sister to a *serpenticola*/*subpinnatifida* clade, *B. lasiocarpa* sister to this core group, and *B. exilis* sister to the rest. The discovery that *B. puberula* consists of two lineages (*arida* and *puberula*) which seem to be able to hybridize, yet maintain a clear distinction regarding their genetic variation, is a novel finding. This distinction was apparent in the PCA, the admixture analyses as well as the evolutionary placement within the *puberula* group (see Figs. 2, 3, 6, and Additional file 1: Figure S4). Based on the microsatellite dataset presented by [43], both lineages of *B. puberula* occur in Oregon, Utah, Idaho, and Nevada, whereas only *B. puberula puberula* has been found in Idaho (Fig. 1). We have not observed any mixed populations but, based on their known distributions and habitat requirements, they are likely to be sympatric somewhere near where 42° N latitude crosses Nevada and Oregon.

When assessing admixture among the presented taxa and clustering into groups, the placement of most individuals corresponded well with the nominal taxa obtained from microsatellites. We did, however, discover apparent admixture among members of the *puberula* group and the more distantly-related taxa included in this study. We were able to find signatures of admixture extending beyond the *B. puberula* group, both with the estimated admixture proportions and when considering the population graph structure inferred by treemix (Figs. 3 and 5). We further found that individuals of *B. stricta*, *B. retrofracta* and *B. exilis* experienced admixture from all members of the *puberula* group. More generally, we observed that all lineages considered in this study were involved in admixture events. Whereas in some cases evidence of admixture could reflect complex patterns of ancestral structure without admixture e.g. samples 39 and 60; [85], this is less likely in cases where admixture is between more distantly related species or when admixed and non-admixed individuals co-occur.

Based on our results, it appears that gene flow/admixture is reduced between more closely-related taxa. This would seem plausible for the taxa that do not occur in sympatry, such as *B. lasiocarpa* and *B. serpenticola* see also [90], but seems rather surprising given the wide geographic distribution of *B. p. puberula*, *B. p. arida*, and *B. subpinnatifida*, and overlapping flowering times among taxa belonging to the *puberula* group [43, 91], combined with otherwise clear signs of admixture in more distantly related taxa (group 1 in Fig. 4). Widespread hybridization has repeatedly been reported in the genus *Boechera* e.g. [42, 53, 55]. However, it appears as if some lineages were more prone to viable hybridization and introgression than others. When considering admixture in sympatry as an integrative measure of RI, we have to acknowledge some limitations. When sampling across large geographical areas, it is possible to miss hybridization events if there are only few individuals with mixed ancestry present. Additionally, it might be possible to encounter early-generation hybrids (predominantly F1s) that do not contribute to either gene pool. Additionally, back-crosses could fail to reproduce, which would maintain complete RI. This study might represent a rather conservative estimate of RI in the *B. puberula* group, since it is likely that we failed to sample additional hybrids, given the wide geographic distribution of the involved taxa.

In triploids that were included in this study, we detected admixture of members of the *B. puberula* group with other taxa, and we assume that those represent apomictic individuals, resulting from hybridization events, because it has been shown that other triploid *Boechera* hybrids predominantly reproduce asexually [53, 72, 92]. This assumption, however, should be tested to ascertain whether it applies to the *B. puberula* group in particular. Additionally, it is not clear whether apomictic individuals introgress into sexual lineages through facultative sexuality [93, 94], or whether these apomicts remain mostly isolated from sexual lineages [95]. Across angiosperm groups, the frequency of polyploids among genera ranges from 29 and 46% [96]. Across our samples of the genus *Boechera*, 26% of individuals were found to be polyploid, indicating a slightly lower frequency of polyploids. However, given the wide geographic sampling, this estimate might not be representative for the entire genus. We have yet to develop reasonable estimates of divergence times within the family Brassicaceae, let alone the genus *Boechera*, due to a poor fossil record [78]. Despite the lack of temporal resolution, our results suggest variable rates of the accumulation of incompatibilities in the genus *Boechera*.

Future projects include formal tests for the accumulation of reproductive barriers between the presented lineages, the examination of individual lineages in this group on a population genetic level, crossing experiments to gain an understanding of pre- and postzygotic isolating barriers and their relative contribution to overall RI, determination of reproductive modes (sexual vs. asexual), and determination of mating system of sexually reproducing individuals [97]. Currently, analyses of *B. lasiocarpa* populations are underway, which will further shed light on the maintenance of genetic variation in this lineage, as well as patterns of admixture with other taxa in this complex genus. Members of the *B. puberula* group represent feasible models for the study of speciation in sympatry, allopatry, and parapatry, adaptation to specific soils (e.g. calciferous and serpentenoid), the roles of reproductive modes and mating system in the maintenance of genetic diversity, as well as chromosomal rearrangements after polyploidization events and whole-genome duplications [98, 99]. Studying these components will bring us closer to understanding the processes that drive speciation as well as the maintenance of genetic diversity in structured populations with differential reproductive modes.

## Conclusions

We present evidence that the *B. puberula* species complex is monophyletic, and that *B. puberula* comprises two lineages. Based on 14,815 common variants, we found evidence for widespread admixture across taxa. Admixture appears to be genetically and geographically widespread and has been occurring for several generations. Further, we found that the accumulation of reproductive isolation between species does not seem to occur linearly with time since divergence in this system.

## Additional file

Additional file 1 Supplementary Figures and Tables. (PDF 658 kb)
