# Supplementary material for: Admixture, evolution, and variation in reproductive isolation in the Boechera puberula clade
Source: BMC Evol Biol. 2018 Apr 25;18:61. doi: 10.1186/s12862-018-1173-6 (PMC5921550; doi:10.1186/s12862-018-1173-6)
Supplement: Supplementary file 1 — Supplementary Figures and Tables. (PDF 658 kb) [file 12862_2018_1173_MOESM1_ESM.pdf]

Admixture, evolutionary relationships, and variation in reproductive isolation in the *Boechera puberula* clade

Additional tables & figures

Martin P. Schilling, Zach Gompert, Fay-Wei Li, Michael D Windham, Paul G. Wolf

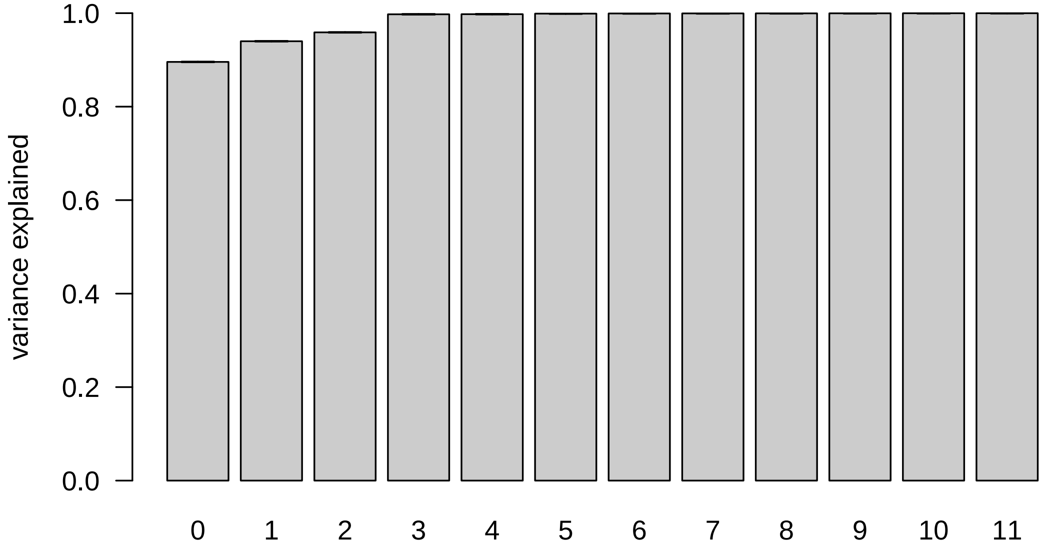

Figure S1: Percentage of variance explained and standard error of treemix runs with m = 0-11

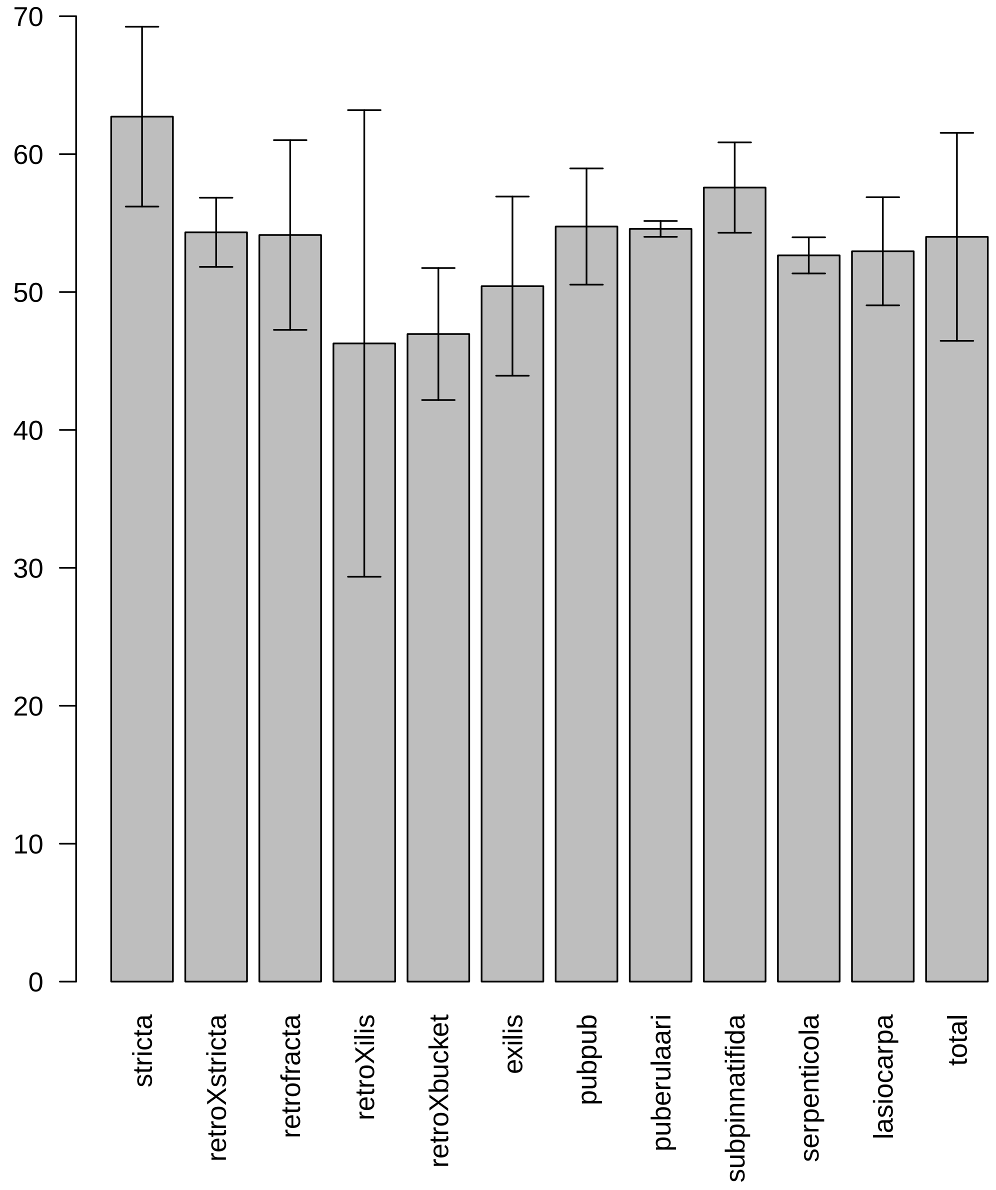

Figure S2: Mean species-specific mapping rates and standard deviation for individuals represented in this study, with groups corresponding to groups used in `treemix` analyses (with sample ids 25, 26, 33, 34 in 'retroXbucket').

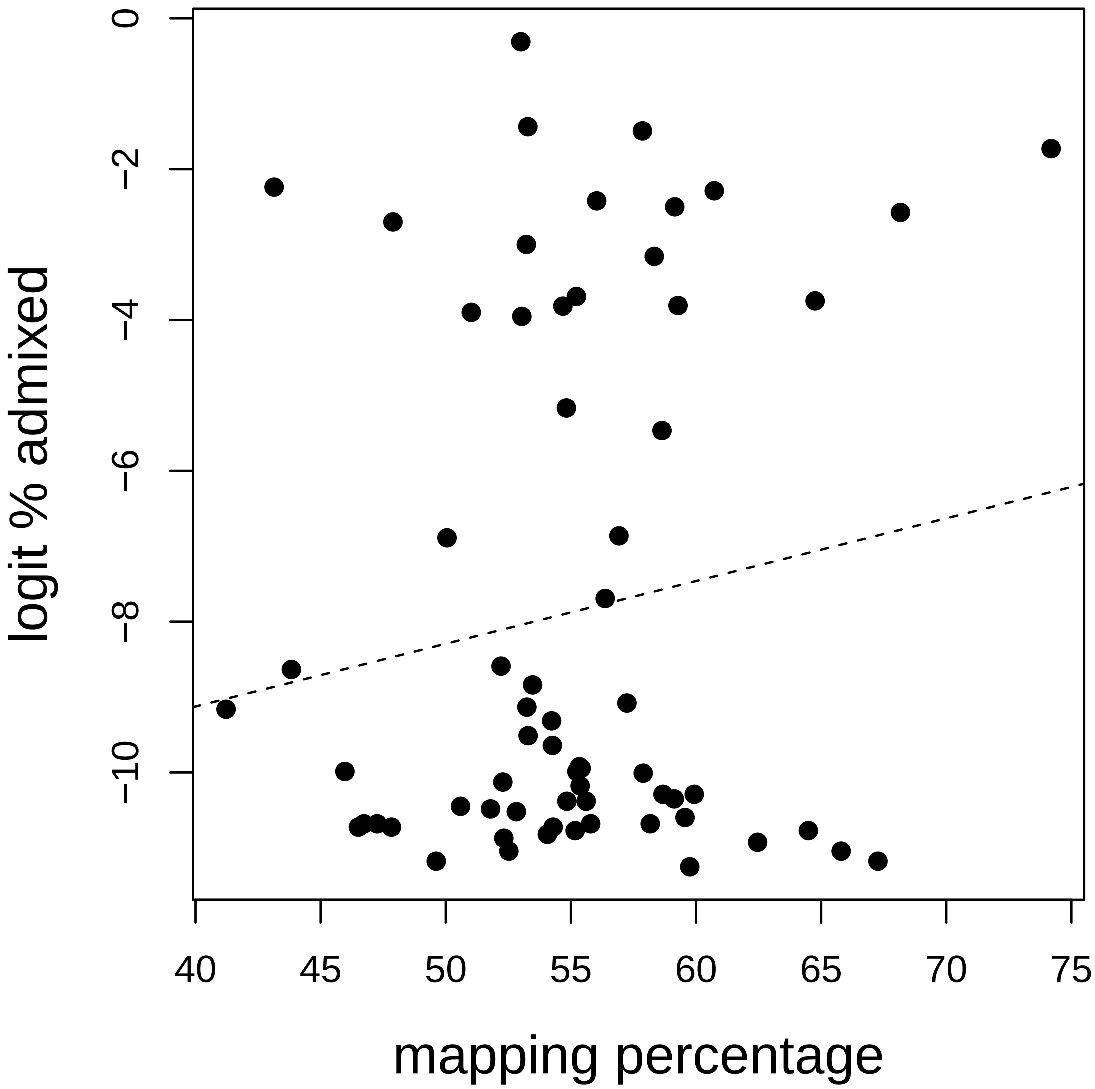

Figure S3: Scatterplot of individual mapping rates and logit percent admixed (i.e. 1 - maximum admixture proportion) for each individual (the best fit line from a linear model is included). We find no evidence that low mapping rates are associated with increased evidence of admixture, but rather a non-significant trend in the oppossite direction ($\beta = 0.083$, P = 0.256).

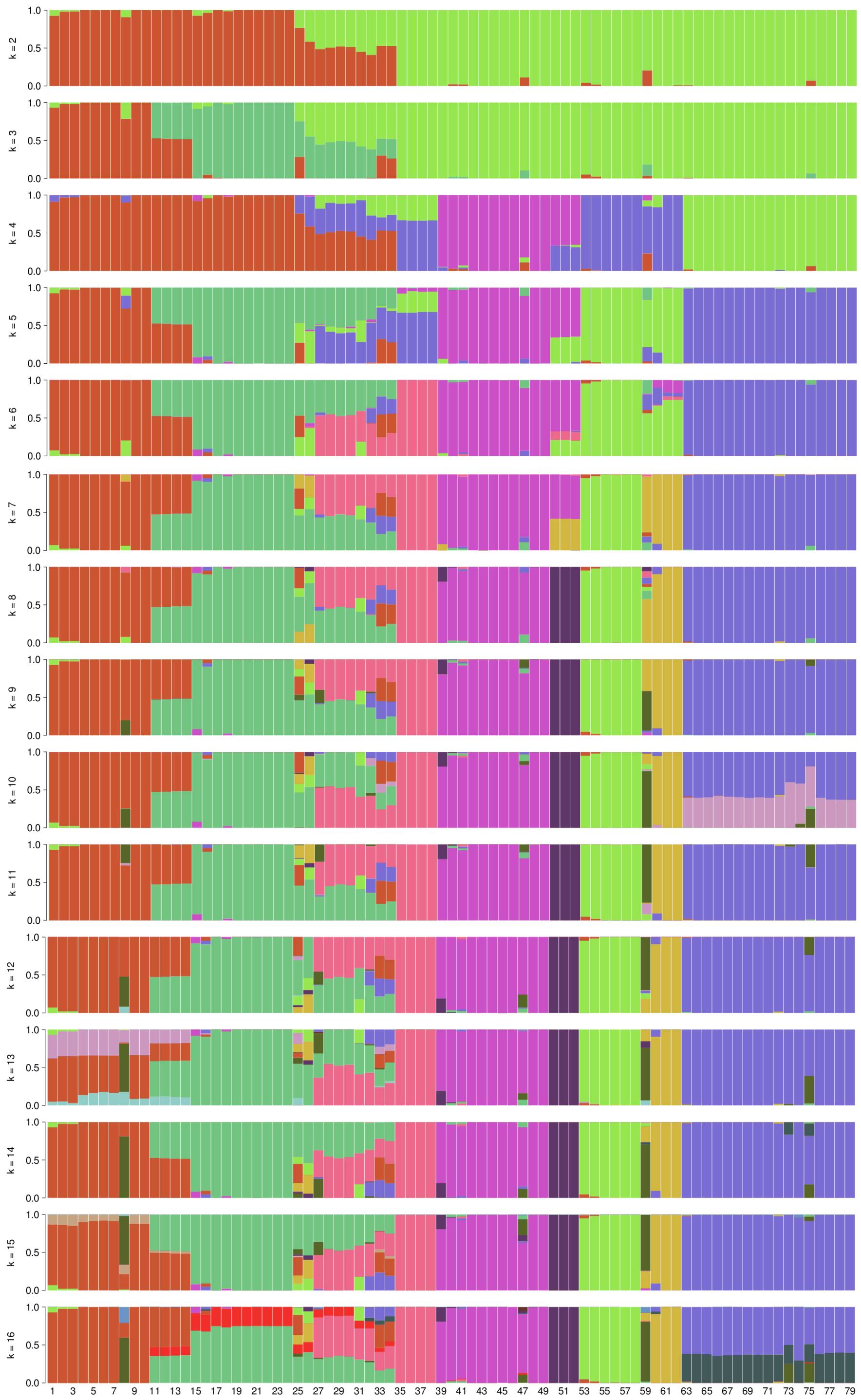

Figure S4: Admixture proportions based on 14,815 common variants. Each bar represents Bayesian point estimates of admixture proportions for each respective individual, and thus the proportion of inheritance of each genome to the respective species. Results of 2 through 16 presumed source species are shown here, with k = 8 being the best model based on DIC. Given the higher number of putative source taxa, we chose a color palette that differs from the colors used in the figures which were included in the paper.

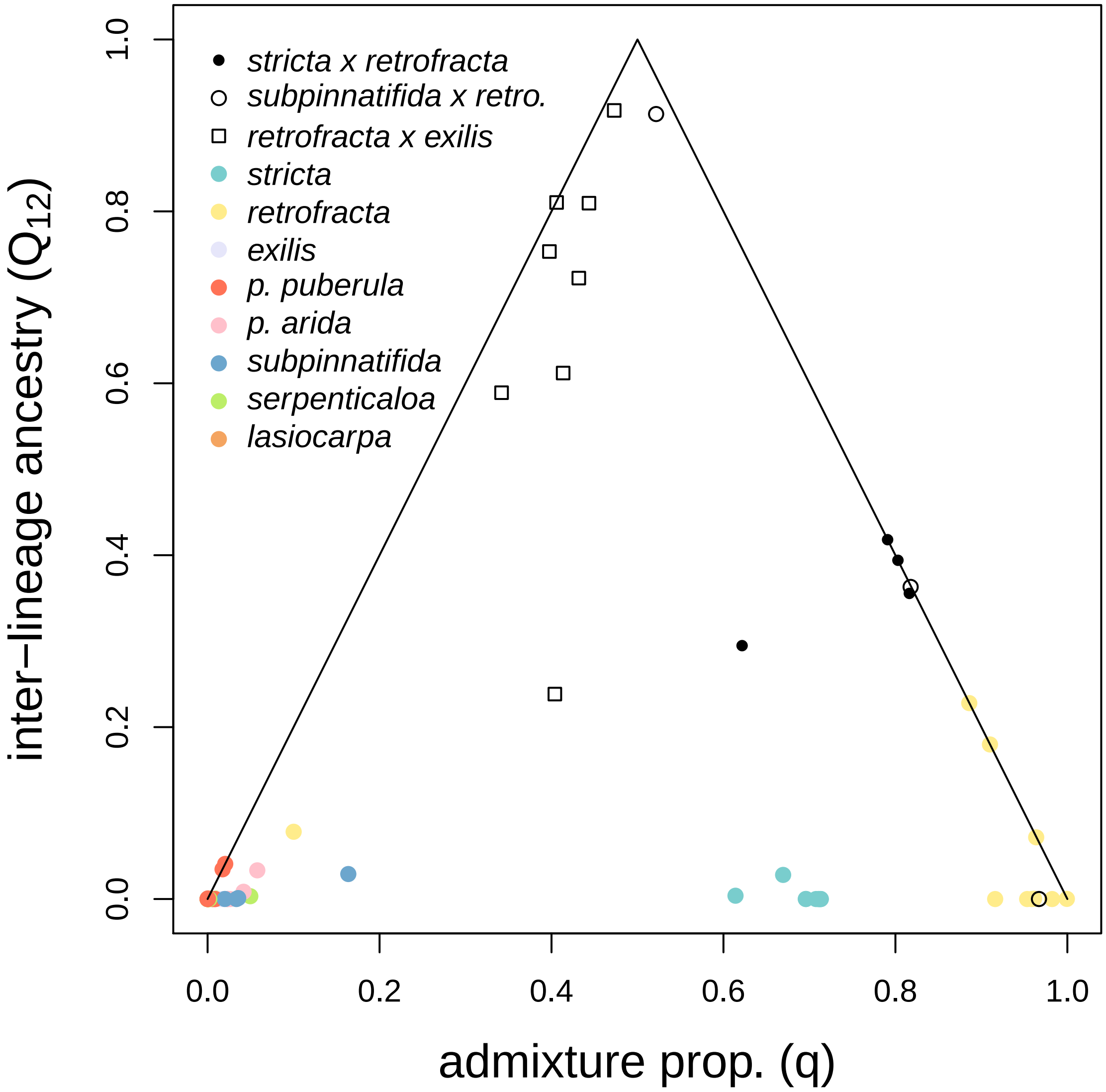

Figure S5: The plot shows the relationship between the admixture proportion q_1 (with k = 2) and inter-lineage ancestry (Q_{12}; i.e., the proportion of the genome where an individual is heterozygous for ancestry). Colored dots denote individuals. Lines indicate the maximum possible inter-source population ancestry given global genetic ancestry (individuals on these line have at least one non-admixed parent)

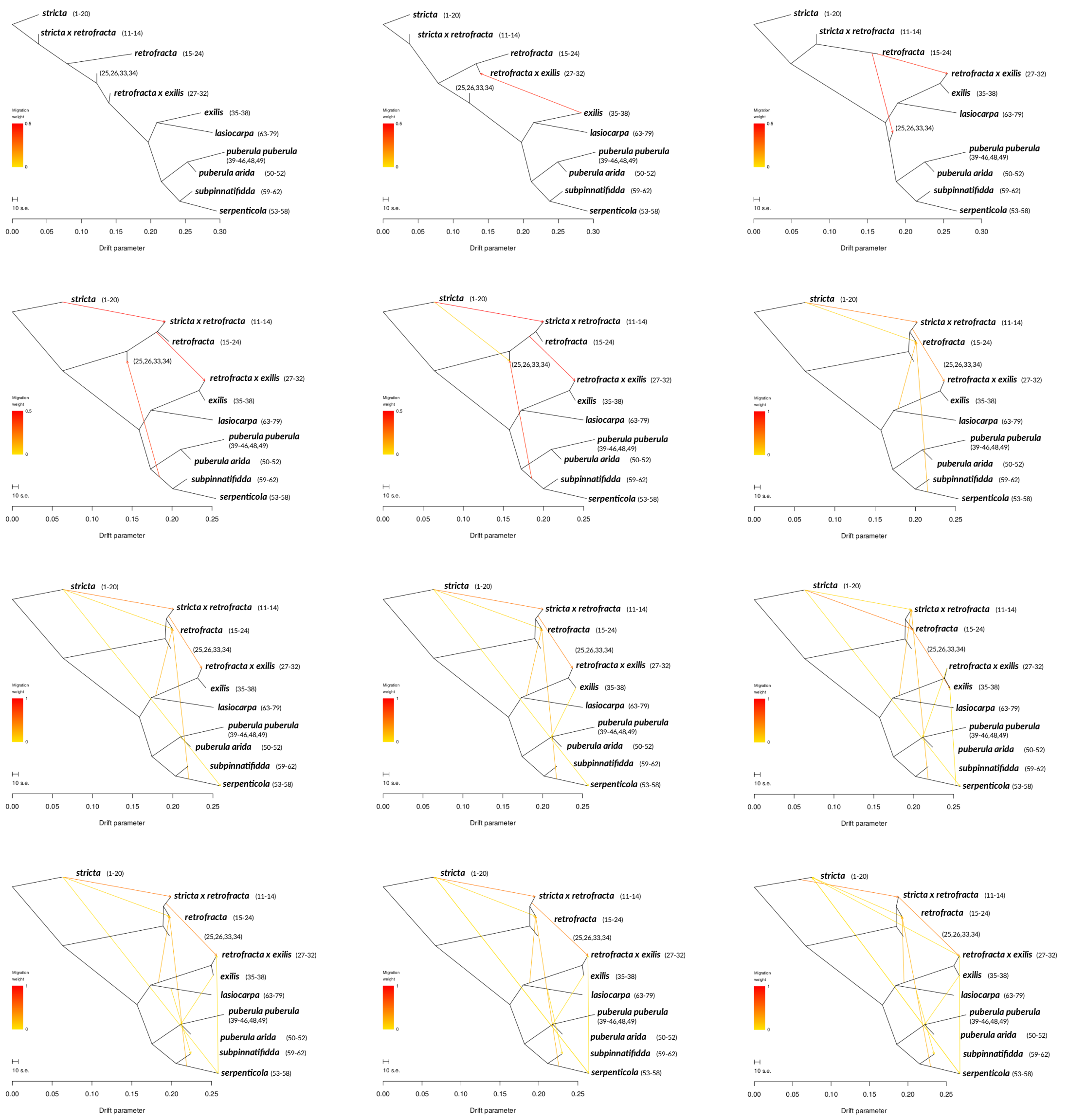

Figure S6: Population graphs inferred by `treemix` for diploid *Boechera* taxa included in this study, allowing 0-11 migration or admixture events. Branch lengths are proportional to genetic drift.

Table S1: Locality information, locality id (locID) sample numbers (id) for Fig. 2, internal id (iID) as well as ploidy and nominal taxa which were both determined from microsatellite data. Here, we use only the species epithet, omitting the genus name. arida = *B. puberula arida*, puberula = *B. puberula puberula*. The 23 samples not denoted with "MS" came from air-dried herbarium specimens

locID	id	iID	locality	longitude	latitude	ploidy	nominal taxon
1	1	MS556	Grizzly Peak, UT	-111.97	41.41	2	*B. stricta*
1	2	MS557	Grizzly Peak, UT	-111.97	41.41	2	*B. stricta*
1	3	MS558	Grizzly Peak, UT	-111.97	41.41	2	*B. stricta*
2	4	CR1181	La Plata, CO	-108.02	37.44	2	*B. stricta*
3	5	JB186	Weber, UT	-111.59	41.41	2	*B. stricta*
4	6	JB377	Nye, NV	-117.35	38.95	2	*B. stricta*
5	7	JB1255	Custer, ID	-114.65	43.86	2	*B. stricta*
1	8	MS554	Grizzly Peak, UT	-111.97	41.41	2	*B. stricta*
6	9	CR1091	Teton, WY	-110.52	43.85	2	*B. stricta*
7	10	JB1258	Madison, MO	-111.96	45.56	2	*B. stricta*
8	11	MS465	Steep Canyon, UT	-111.6	41.97	2	*B. retrofracta x stricta*
9	12	MS328	Deadfall Lake, CA	-122.52	41.33	2	*B. retrofracta x subpinnatifida*
8	13	MS469	Steep Canyon, UT	-111.6	41.97	2	*B. retrofracta x stricta*
8	14	MS471	Steep Canyon, UT	-111.6	41.97	2	*B. retrofracta x stricta*
10	15	MS169	Little Volcano, CA	-120.89	39.86	2	*B. retrofracta*
11	16	MS280	Hat Creek, CA	-121.41	40.7	2	*B. retrofracta*
12	17	JB867	Park, WY	-110.57	44.41	2	*B. retrofracta retrofracta* (sexual)
10	18	MS163	Little Volcano, CA	-120.89	39.86	2	*B. retrofracta*
10	19	MS165	Little Volcano, CA	-120.89	39.86	2	*B. retrofracta*

Continued on next page

Table S1 – *Continued from previous page*

locID	id	iID	locality	longitude	latitude	ploidy	nominal taxon
13	20	JB176	Mineral, MO	-115.7	47.45	2	*B. retrofracta retrofracta* (sexual)
11	21	MS282	Hat Creek, CA	-121.41	40.7	2	*B. retrofracta*
14	22	JB659	Deschutes, OR	-121.56	43.67	2	*B. retrofracta retrofracta* (sexual)
15	23	CR1043	Humboldt, CA	-123.65	40.48	2	*B. retrofracta retrofracta* (sexual)
9	24	MS313	Deadfall Lake, CA	-122.51	41.33	2	*B. retrofracta*
8	25	MS473	Steep Canyon, UT	-111.6	41.97	2	*B. retrofracta x stricta*
9	26	MS327	Deadfall Lake, CA	-122.52	41.33	2	*B. retrofracta x subpinnatifida*
16	27	MS413	Bear Lake Summit, UT	-111.47	41.93	2	*B. exilis x retrofracta*
17	28	MS9	Wells, NV	-114.57	41.08	2	*B. exilis x retrofracta*
17	29	MS7	Wells, NV	-114.57	41.08	2	*B. exilis x retrofracta*
17	30	MS18	Wells, NV	-114.57	41.08	2	*B. exilis x retrofracta*
16	31	MS425	Bear Lake Summit, UT	-111.47	41.93	2	*B. exilis x retrofracta*
17	32	MS11	Wells, NV	-114.57	41.08	2	*B. exilis x retrofracta*
16	33	MS426	Bear Lake Summit, UT	-111.47	41.93	2	*B. exilis x retrofracta*
16	34	MS422	Bear Lake Summit, UT	-111.47	41.93	2	*B. exilis x retrofracta*
18	35	FW241	Elko, NV	-115.08	40.68	2	*B. exilis*
19	36	CR1164	Summit, UT	-111.4078	40.7753	2	*B. exilis*
20	37	FW73	Nye, NV	-117.54	38.97	2	*B. exilis*
21	38	FW76	Millard, UT	-112.27	38.95	2	*B. exilis*
22	39	JB1275	Baker, OR	-117.11	44.7	2	*B. puberula puberula*
23	40	MS79	Water Canyon, NV	-116.71	40.64	2	*B. puberula*
23	41	MS80	Water Canyon, NV	-116.71	40.64	2	*B. puberula*
24	42	JB382	Box Elder, UT	-113.94	41.77	2	*B. puberula puberula*
25	43	MS93	Lye Creek, NV	-117.54	41.69	2	*B. puberula*
26	44	JB381	Humboldt, NV	-117.55	41.67	2	*B. puberula puberula*
25	45	MS102	Lye Creek, NV	-117.54	41.69	2	*B. puberula*
25	46	MS94	Lye Creek, NV	-117.54	41.69	2	*B. puberula*

Continued on next page

Table S1 – *Continued from previous page*

locID	id	iID	locality	longitude	latitude	ploidy	nominal taxon
11	47	MS283	Hat Creek, CA	-121.41	40.7	2	*B. retrofracta*
23	48	MS69	Water Canyon, NV	-116.71	40.64	2	*B. puberula*
23	49	MS72	Water Canyon, NV	-116.71	40.64	2	*B. puberula*
27	50	JB1611	Box Elder, UT	-113.69	41.53	2	*B. puberula arida*
28	51	JB1610	Mono, CA	-119.13	38.36	2	*B. puberula arida*
29	52	FW237	Lander, NV	-117.37	39.24	2	*B. puberula arida*
30	53	MS286	Bully Choop Mtn, CA	-122.94	40.65	2	*B. serpenticola*
30	54	MS305	Bully Choop Mtn, CA	-122.94	40.65	2	*B. serpenticola*
30	55	MS302	Bully Choop Mtn, CA	-122.94	40.65	2	*B. serpenticola*
30	56	MS294	Bully Choop Mtn, CA	-122.94	40.65	2	*B. serpenticola*
30	57	MS298	Bully Choop Mtn, CA	-122.94	40.65	2	*B. serpenticola*
30	58	MS297	Bully Choop Mtn, CA	-122.94	40.65	2	*B. serpenticola*
31	59	MS367	Rogue River, OR	-123.53	42.55	2	*B. subpinnatifida*
31	60	MS392	Rogue River, OR	-123.53	42.55	2	*B. subpinnatifida*
31	61	MS402	Rogue River, OR	-123.53	42.55	2	*B. subpinnatifida*
31	62	MS403	Rogue River, OR	-123.53	42.55	2	*B. subpinnatifida*
32	63	FW516	Rich, UT	-111.46	41.92	2	*B. lasiocarpa* (holotype)
33	64	CR1308	Cache, UT	-111.71	41.8	2	*B. lasiocarpa*
33	65	CR1309	Cache, UT	-111.66	41.91	2	*B. lasiocarpa*
34	66	MS458	Logan Canyon Sinks, UT	-111.48	41.93	2	*B. lasiocarpa*
34	67	MS444	Logan Canyon Sinks, UT	-111.48	41.93	2	*B. lasiocarpa*
34	68	MS446	Logan Canyon Sinks, UT	-111.48	41.93	2	*B. lasiocarpa*
34	69	MS455	Logan Canyon Sinks, UT	-111.48	41.93	2	*B. lasiocarpa*
34	70	MS453	Logan Canyon Sinks, UT	-111.48	41.93	2	*B. lasiocarpa*
34	71	MS447	Logan Canyon Sinks, UT	-111.48	41.93	2	*B. lasiocarpa*
35	72	MS485	Steam Mill Peak, UT	-111.61	41.95	2	*B. lasiocarpa*
35	73	MS496	Steam Mill Peak, UT	-111.61	41.95	2	*B. lasiocarpa*

Continued on next page

Table S1 – *Continued from previous page*

locID	id	iID	locality	longitude	latitude	ploidy	nominal taxon
35	74	MS488	Steam Mill Peak, UT	-111.61	41.95	2	*B. lasiocarpa*
11	75	MS275	Hat Creek, CA	-121.41	40.7	2	*B. retrofracta*
36	76	JB419	Box Elder, UT	-111.98	41.39	2	*B. lasiocarpa*
37	77	MS513	James Peak, UT	-111.78	41.38	2	*B. lasiocarpa*
38	78	JB1587	Salt Lake, UT	-111.72	40.63	2	*B. lasiocarpa*
39	79	FW1630	Tooele, UT	-112.62	40.48	2	*B. lasiocarpa*
40	NA	MS39	Angel Lake, NV	-115.07	41.02	3	*B. exilis x puberula x retrofracta*
40	NA	MS40	Angel Lake, NV	-115.07	41.02	3	*B. exilis x puberula x retrofracta*
40	NA	MS41	Angel Lake, NV	-115.07	41.02	3	*B. exilis x puberula x retrofracta*
41	NA	MS215	Frenchman Lake, CA	-120.19	39.88	3	*B. p. arida x puberula x subpinnatifida*
41	NA	MS221	Frenchman Lake, CA	-120.18	39.87	3	*B. retrofracta x sparsiflora* (2:1)
41	NA	MS228	Frenchman Lake, CA	-120.18	39.87	3	*B. retrofracta x sparsiflora* (2:1)
42	NA	MS125	Indian Creek, NV	-117.55	41.65	3	*B. exilis x retrofracta x sparsiflora*
42	NA	MS128	Indian Creek, NV	-117.55	41.65	3	*B. exilis x retrofracta x sparsiflora*
42	NA	MS134	Indian Creek, NV	-117.55	41.65	3	*B. exilis x retrofracta x sparsiflora*
42	NA	MS135	Indian Creek, NV	-117.55	41.65	3	*B. exilis x retrofracta x sparsiflora*
43	NA	MS501	James Peak, UT	-111.78	41.38	3	*B. retrofracta x lasiocarpa* (2:1)
43	NA	MS511	James Peak, UT	-111.78	41.38	3	*B. retrofracta x lasiocarpa* (2:1)
43	NA	MS512	James Peak, UT	-111.78	41.38	3	*B. retrofracta x lasiocarpa* (2:1)
43	NA	MS525	James Peak, UT	-111.78	41.38	3	*B. retrofracta x lasiocarpa* (2:1)
44	NA	MS145	Peavine Peak, NV	-119.93	39.59	3	*B. p. arida x subpinnatifida* (2:1)
44	NA	MS150	Peavine Peak, NV	-119.93	39.59	3	*B. p. arida x subpinnatifida* (2:1)
44	NA	MS162	Peavine Peak, NV	-119.93	39.59	3	*B. p. arida x subpinnatifida* (2:1)
45	NA	MS47	Shoshone Mtns, NV	-116.86	40.42	3	*B. exilis x puberula x retrofracta*
45	NA	MS49	Shoshone Mtns, NV	-116.86	40.42	3	*B. exilis x puberula x retrofracta*
45	NA	MS54	Shoshone Mtns, NV	-116.86	40.42	3	*B. exilis x puberula x retrofracta*
46	NA	MS482	Steam Mill Peak, UT	-111.61	41.95	3	*B. lasiocarpa x retrofracta x stricta*

Continued on next page

Table S1 – *Continued from previous page*

locID	id	iID	locality	longitude	latitude	ploidy	nominal taxon
46	NA	MS499	Steam Mill Peak, UT	-111.61	41.95	3	*B. lasiocarpa x retrofracta x stricta*
47	NA	MS536	Willard Peak, UT	-111.97	41.39	3	*B. lasiocarpa x lemmonii x stricta*
47	NA	MS538	Willard Peak, UT	-111.97	41.39	3	*B. lasiocarpa x lemmonii x stricta*
47	NA	MS539	Willard Peak, UT	-111.97	41.39	3	*B. lasiocarpa x lemmonii x stricta*
47	NA	MS543	Willard Peak, UT	-111.97	41.39	3	*B. lasiocarpa x lemmonii x stricta*
47	NA	MS550	Willard Peak, UT	-111.97	41.39	3	*B. lasiocarpa x lemmonii x stricta*
47	NA	MS552	Willard Peak, UT	-111.97	41.39	3	*B. lasiocarpa x lemmonii x stricta*
